# Glucose-regulated protein 78 substrate-binding domain alters its conformation upon EGCG inhibitor binding to nucleotide-binding domain: Molecular dynamics studies

**DOI:** 10.1038/s41598-018-22905-6

**Published:** 2018-04-03

**Authors:** K. R. D. Sagara N. S. Gurusinghe, Aanchal Mishra, Seema Mishra

**Affiliations:** 1 Department of Biochemistry, School of Life Sciences, University of Hyderabad, Hyderabad, Telangana India; 2 Ronin Institute for Independent Scholarship, Montclair, NJ USA; 3 Osmania University, Hyderabad, Telangana India

## Abstract

Glucose-regulated protein 78 (GRP78), is overexpressed in glioblastoma, other tumors and during viral and bacterial infections, and so, it is postulated to be a key drug target. EGCG, an ATP-competitive natural inhibitor, inhibits GRP78 effect in glioblastoma. Structural basis of its action on GRP78 nucleotide-binding domain and selectivity has been investigated. We were interested in exploring the large-scale conformational movements travelling to substrate-binding domain *via* linker region. Conformational effects of EGCG inhibitor as well as ATP on full length GRP78 protein were studied using powerful MD simulations. Binding of EGCG decreases mobility of residues in SBDα lid region as compared to ATP-bound state and similar to apo state. The decreased mobility may prevent its opening and closing over SBDβ. This hindrance to SBDα subdomain movement, in turn, may reduce the binding of substrate peptide to SBDβ. EGCG binding folds the protein stably as opposed to ATP binding. Several results from EGCG binding simulations are similar to that of the apo state. Key insights from these results reveal that after EGCG binding upon competitive inhibition with ATP, GRP78 conformation may revert to that of inactive, apo state. Further, SBD may adopt a semi-open conformation unable to facilitate association of substrates.

## Introduction

Gliomas (including ependymomas, glioblastomas, and oligodendrogliomas) are a heterogenous bunch of tumors which represent thirty percent of the central nervous system tumors. Among these, glioblastomas (GBM) are a type of highly malignant astrocytic tumors characterized by rapid progression^1^. These brain tumors were also given the names glioblastoma multiforme and astrocytoma (tumors that arise from astrocytes). WHO has classified GBM in grade IV (highest grade) category because of its highly malignant form and, therefore, research in GBM is among the highest funded by the National Institutes of Health (NIH)^2–4^. Like many other tumor types, exact cause for GBM development as well as progression is not yet discovered^5^. Till date, several treatments options such as chemotherapy with temozolomide, surgery and radiotherapy have been implemented, but with limited success.

Glucose regulated protein 78 (GRP78), also known as Binding Immunoglobulin Protein (BiP) is overexpressed in glioblastoma independent of p53 and PTEN status^6^. This protein belongs to HSP70 family of proteins, expressed in endoplasmic reticulum and is a stress-induced protein involved in the Unfolded Protein Response (UPR), for proper folding of the proteins synthesized by the ribosomes^7–9^. It was found that tumors with rapid growth rate and aggressive property showed an overexpression of GRP78. It is also found overexpressed in several other tumor types as well and postulated to be a universal drug target for Ebola virus, influenza virus and hepatitis virus infections. Expression of GRP78 is inversely proportional to the survival of the patient and can be used as a predictive factor. GRP78 is also involved in the regulation of apoptotic pathways. It has been found that this protein has the ability to form an antiapoptotic complex in the endoplasmic reticulum complexing with caspase7. This, in turn, reduces the activation of caspase7. This leads to glioma cells becoming resistant to etoposide- and cisplatin-induced apoptosis^10^. Controlling the expression of GRP78, therefore, serves as an important approach in targeting glioblastoma^11^.

The structure of GRP78 (see Supplementary Fig. S1) mainly consists of a nucleotide-binding domain (NBD), and a substrate-binding domain (SBD). NBD binds ATP while SBD binds substrate peptide/protein in the form of excluded segment or partially folded protein^12^. These two main domains are connected by an inter domain linker which is highly conserved. GRP78 protein consists of a total of 633 amino acid residues. NBD comprises of residues 25–408, with a linker region of residues 408–419 connecting NBD with residues 419–633 belonging to the SBD. NBD is made up of two lobes, lobe I subdivided into two subdomains known as IA and IB and lobe II further subdivided into IIA and IIB. SBD is also divided into two subdomains known as SBDα, a 10 kDa subdomain with residues from 529–633 and SBDβ, a 25 kDa subdomain with residues from 419–529. SBDα subdomain acts as a lid which covers SBDβ in a manner which facilitates tight binding of the substrate. SBDβ binds to NBD in such a way that it is connected to both the lobes of NBD, while SBDα docks on lobe I side of NBD and the first two helices are fused into one long helical form.

During the functioning of GRP78, it undergoes two major conformational changes. These conformations are termed as open and closed conformation (see Supplementary Fig. S2). Open conformation is the ATP-bound state, where this nucleotide binds to the nucleotide binding domain^13^. Closed conformation is the ADP-bound state, where there is only a little interaction between the two main domains (NBD and SBD).

Generally GRP78 is in ATP-bound state (bound in a reversible manner). One important characteristic of GRP78 protein is that, NBD domain itself has a weak ATPase activity not sufficient to fully hydrolyse ATP. Due to this, spontaneous hydrolysis does not occur. When proteins or polypeptides are released from ribosomes after translation, these will be recognized by the substrate binding domain. SBD recognizes short degenerative sequence enriched in hydrophobic amino acid core (five residues). Binding of the peptide substrate increases the rate of ATP hydrolysis. This leads to closing of binding pocket in SBD, tightly binding the peptide, this is termed a closed conformation, while ADP is simultaneously bound to NBD. SBDα is in such a position that it covers the substrate bound to SBDβ. As the result of this covering, association and dissociation rates of the substrate get reduced. Hence, there will be an excessive high affinity between the substrate and the substrate binding domain.

In open conformation, structural analyses have shown that the angle between SBDα and SBDβ, and between strands 1 and 4 of latter SBD, is much larger and wider than that of the closed conformation^13^, (see Supplementary Fig. S2). As a result, the association and dissociation rates of peptide substrates are high. Consequently, SBD will have a low affinity towards the substrate (i.e., substrate is not bound). Hydrolysis of ATP helps in further transition of open conformation to closed conformation. After the full protein/peptide substrate has been folded, nucleotide exchange factors such as BAG-1 and HspBP1, stimulate ADP release and ATP binding and again shifting to open conformation, so that the protein can undergo another round of its function. The cycle is then repeated.

In closed conformation (see Supplementary Fig. S2), the structure of full protein looks like that of isolated structures of NBD and SBD^13^. NBD of open conformation shows a drastically different form with a rotation of lobe I relative to lobe II, resulting in opening of nucleotide-binding cavity. This suggests that, SBD and its interactions with NBD are the key driving forces in the stabilization and inter-conversion of different conformations.

Natural inhibitor (−)-Epigallocatechin gallate **(**EGCG) with a molecular weight of 458.375 g/mol, is a polyphenolic bioflavanoid derived from a variety of plants. It is an epigallocatechin and gallic acid ester (see Supplementary Fig. S3), and is found in high content in the dried leaves of tea comprising of green, white and black tea^14^. EGCG binds to the NBD of GRP78, thereby acting as a competitive inhibitor to ATP, inhibiting the ATPase activity of GRP78^15^. EGCG binding to GRP78 renders the monomer form of GRP78 which is the active state get converted into dimeric and oligomeric inactive forms^16^. EGCG also has the ability to prevent the formation of GRP78-caspase7 complex in the endoplasmic reticulum and this complex is anti-apoptotic. This induces apoptosis in cancer cells. Molecular docking and molecular dynamics studies have revealed the conformational changes of ATPase domain upon EGCG and chemical inhibitor OSU-03012 (AR-12) binding, with EGCG binding more specific to GRP78 than OSU-03012 binding^11^. Previous observations from our published paper^11^ show that after binding of EGCG, unfolded form of GRP78 NBD domain is converted into a folded form, and it is a gradual process. Further, we had also predicted that OSU-03012 binding also alters the conformation as it seems to convert GRP78 NBD unfolded form immediately to a folded form. Hydrogen bonding, electrostatic and hydrophobic interactions with both EGCG and OSU-03012 have also been mapped with important groups, e.g., with gallate moiety of EGCG (in pi-alkyl hydrophobic interactions with Arg297 and Arg367) and phenanthrene group of OSU-03012 (in hydrophobic interaction with Ile61). It is pertinent to mention that a group performing modeling studies using OSU-03012^17^ has misinterpreted our previous results (11) and we had shown that phenanthrene group of OSU-030112 does, indeed, interact with GRP78. The effect of EGCG and OSU-03012 binding to NBD and consequent conformational changes in SBD of full length GRP78 is yet to be studied.

In light of all the information mentioned above, we were interested in understanding structural basis of EGCG action on full-length GRP78 protein. More specifically, the long-range effect of its binding on SBD domain opening and closing to facilitate substrate peptide binding and thereby, functioning of GRP78, is a matter of great interest. The availability of full length crystallographic structure of GRP78 has made these investigations possible. An exploration into how EGCG binding to ATPase domain affects SBD movements and conformational changes, is bound to provide a clear structural basis and mechanistic insights into its mode of action.

## Materials and Methods

### Structure Retrieval

Three dimensional structure, with PDB ID: 5E84 and 5E85 of full length ATP-bound state of 78-kDa Glucose-regulated Protein (GRP78) and its isolated SBD, respectively, was downloaded from Research Collaboratory in Structural Bioinformatics Protein Data Bank (RCSB PDB) (http://www.rcsb.org/pdb/home.do). EGCG inhibitor structure was downloaded from PubChem database in .sdf format. OpenBabel (http://openbabel.org/wiki/main_page) was used to convert this file into .pdb format as an input to AutoDock Vina docking tool.

### Molecular Docking

Molecular docking studies using protein (energy minimized), ATP and EGCG inhibitor were done using AutoDock Vina^18^ protein-ligand docking tool within the PyRx 0.8 virtual screening tool. Docked structures with ATP were compared with crystal structures to provide benchmarking. Docking parameters were selected by comparing these docked structures to the original crystallographic structure (PDB ID: 5E84) and finding out similar binding modes. On the basis of this result, modified parameters for Vina search space were as follows: x-centering: −0.7101, y-centering: 33.96 and z-centering: −28.62. The free-energy scoring function in AutoDock Vina uses information from both knowledge-based potentials and empirical scoring functions, and Broyden-Fletcher-Goldfarb-Shanno (BFGS) algorithm for local optimization. Iterated Local Search optimizer is used for global optimization. ATP and EGCG were docked into ATPase domain (NBD) of full-length GRP78 protein.

### Molecular Dynamics (MD) Simulations

Molecular dynamics simulations were performed using GROMACS 4.6.3 MD simulation package^19^. United-atom GROMOS96 43A1 force field was used for all the three structure simulation procedures. As the starting point, crystal structure and docked structure of GRP78 protein alone, GRP78 with ATP and EGCG inhibitor were used as inputs. The ligand .pdb file was uploaded to PRODRG 2.5 server and the program was run with zero chirality, full charges and with energy minimization parameters selected. Finally, the ligand topology file was generated. To neutralize the total charge of the system, 16Na + counter ions were added using genion command. It was ensured that the system had no steric clashes and so, 1000 steps of steepest descent were used for energy minimization. After energy minimization step, position restraints were applied to both protein and inhibitor. Consequently, equilibration was conducted in two phases. The first phase was conducted under NVT ensemble (isothermal-isochoric) and the second phase was conducted under NPT ensemble (isothermal-isobaric). NVT equilibration was done at 300 K and 100 ps of runtime. NPT equilibration was done with 100 ps of runtime, and Parrinello-Rahman barostat was applied at 1 bar reference pressure. After the system was well-equilibrated, position restraints were released and the production MD was run for 50 ns. 300 K of constant temperature and 1 bar of constant pressure with integration time of 2 fs were used. All the bond lengths were constrained using LINCS algorithm. Particle-mesh Ewald algorithm was used for long-range electrostatic interactions. 1.4 nm was set as the cutoffs for short-range electrostatic and van der Waals interaction.

PyMol and BIOVIA Discovery Studio, XMGRACE plotting tool and GROMACS analysis tools were used for the visualization and analyses of trajectories.

## Results and Discussion

### Molecular Docking

Molecular docking of full-length GRP78 (PDB ID: 5E84) and ATP was done using with AutoDock Vina with modified parameters in order to reproduce the crystallographic orientation and pose. Having obtained near correct orientation, these same docking parameters were used to dock EGCG into the NBD binding site, replacing ATP.

**Figure 1:**
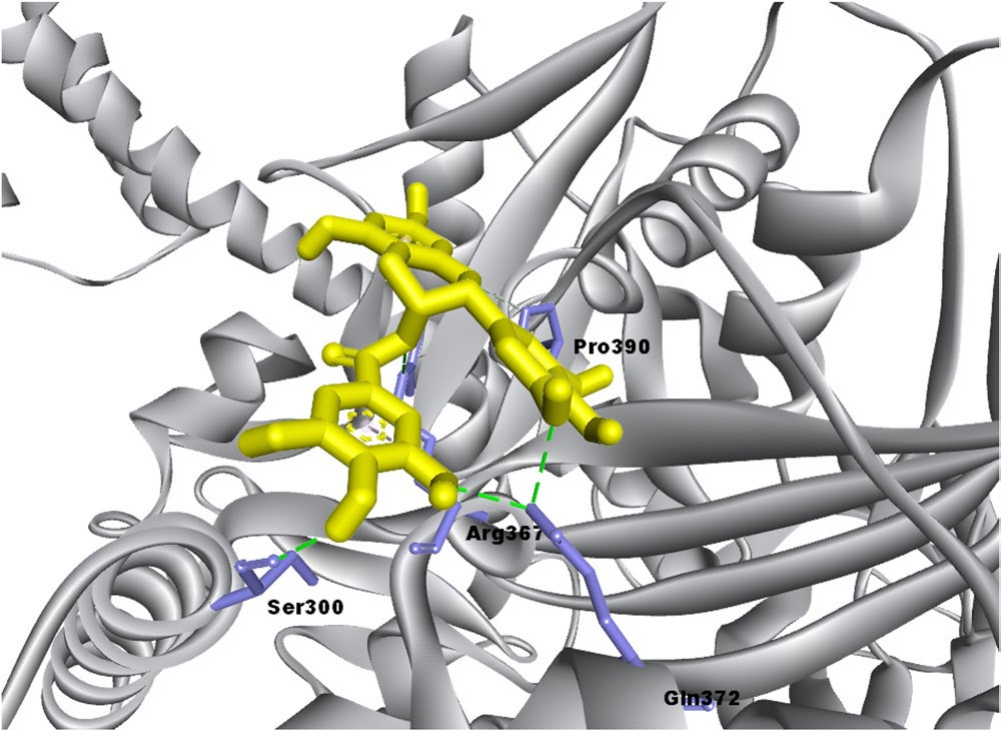
EGCG is shown docked into NBD (ATPase) domain of full length GRP78 using AutoDock Vina. The interacting residues are labelled and shown as light purple sticks while EGCG is shown in yellow ball and stick representation. Interactions are highlighted as follows: hydrogen bonds (green dashed lines), electrostatic (orange dashed lines) and hydrophobic (pink dashed lines) interactions.

**Figure 2:**
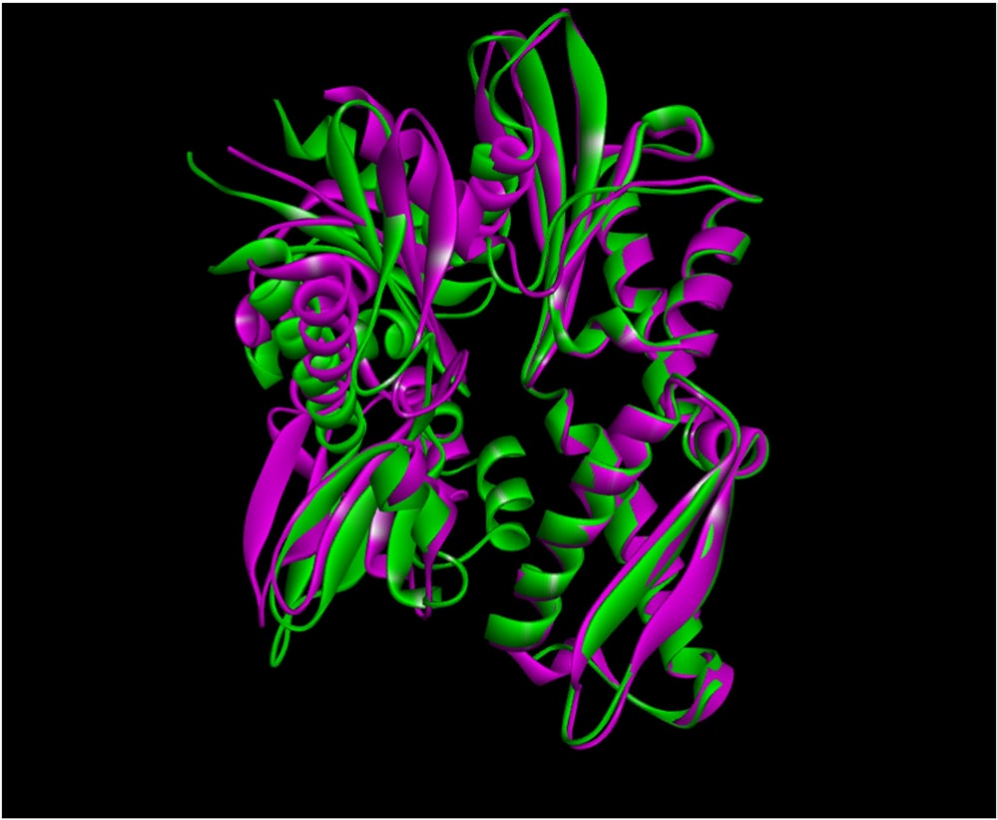
Superimposed crystal structures of NBD from two different PDB files, ID: 3LDL (in purple color) and 5E84 (in green colour).

EGCG docked to full-length GRP78 with a binding energy of −9.9 kcal/mol for the lowest energy docked structure (Fig. 1). Active site residues forming hydrogen bonds with EGCG are: Ser300, Arg367 (2 hydrogen bonds), GLN 372 (three hydrogen bonds) and Pro390. Other residues are involved in non-bonded contacts with the inhibitor. Arg367, in addition to hydrogen bonds, also shows electrostatic interactions with EGCG. EGCG gallate moiety is in hydrophobic interactions with Arg367 as opposed to our earlier studies in which Glu293 is found to be involved in electrostatic interactions with this important moiety of EGCG. This may be due to differences in structures from two different PDB files taken in our studies: PDB ID 5E84 which codes for full-length protein for this work and 3LDL which codes for only the ATPase domain taken due to unavailability of full length structure in previous work. In fact, superimposition of ATPase domain (Fig. 2, green colored structure is from PDB ID: 5E84 while purple colored one is taken from 3LDL) from these two files clearly shows major differences in half of the structure. Domain movements due to the presence of SBD may be one of the cause. In any case, it is clear that the gallate moiety of EGCG is an essential part of its inhibitory activity since it participates in interactions with unique residues of GRP78 in one way or the other^11,15^.

### Molecular Dynamics Simulations

The apo state of GRP78, GRP78-ATP and GRP78-EGCG docked complexes were simulated using GROMACS version 4.6.3 for 50 ns. Having achieved a reasonable equilibrium, the results of these simulations are compared using a wide range of parameters and measurements and are detailed as follows:

#### Stability and Residue fluctuations

**Figure 3:**
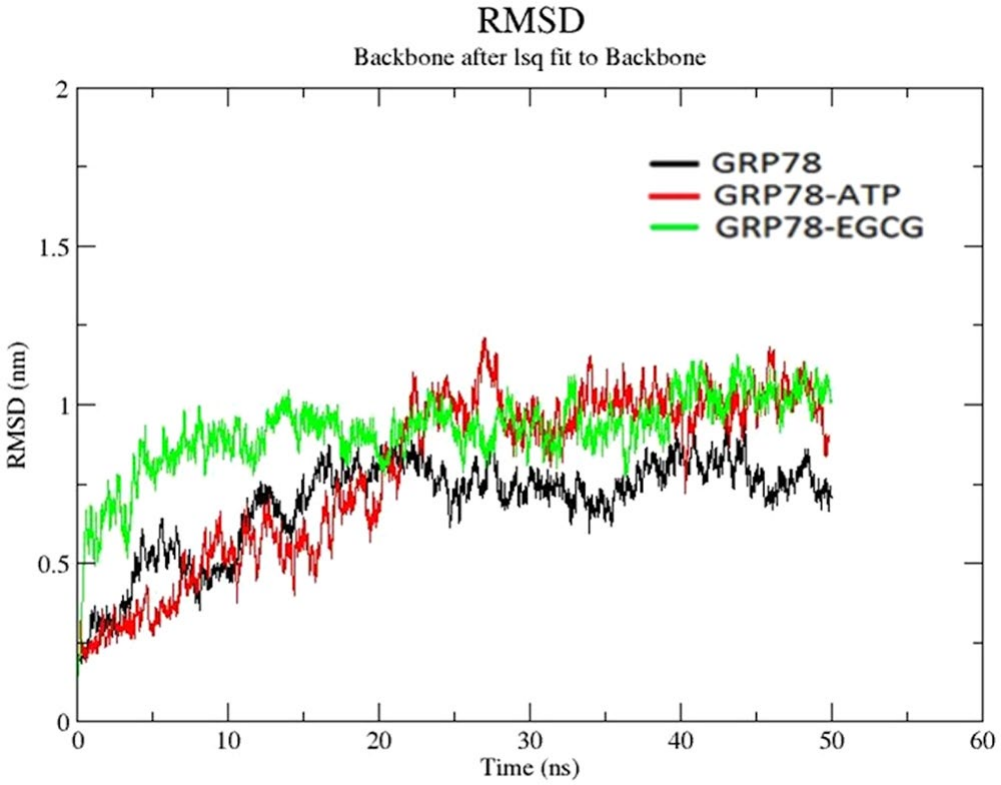
Root mean-square deviation (RMSD) *vs.* MD simulation time plot of the Cα backbone atoms of apo GRP78 protein (black), ATP-bound system (red) and EGCG-bound system (green).

To check if there are any structural changes, RMSD of final structure relative to energy minimized crystal structure and docked structure was calculated for Cα backbone atoms (Fig. 3). As observed from the graph, the apo state of the protein attained a stable conformation after 20 ns. At the beginning, it shows an increase in RMSD value (until 0.8 nm), but after 20 ns it shows a stable value in the range of 0.7–0.9 nm. Binding of ATP shows similar kind of conformational change. It attained a stable conformation after 25 ns in the range of 0.9–1.1 nm. Binding of EGCG stabilized the protein in much less time than apo state and the complex with ATP. The simulation arrives at stable RMSD value after 10 ns in the range of 0.9–1 nm. This gives us an insight into the binding effects of EGCG, first: it helps to stabilize the protein and second: conformational changes occur that are initially very different from the other two.

**Figure 4:**
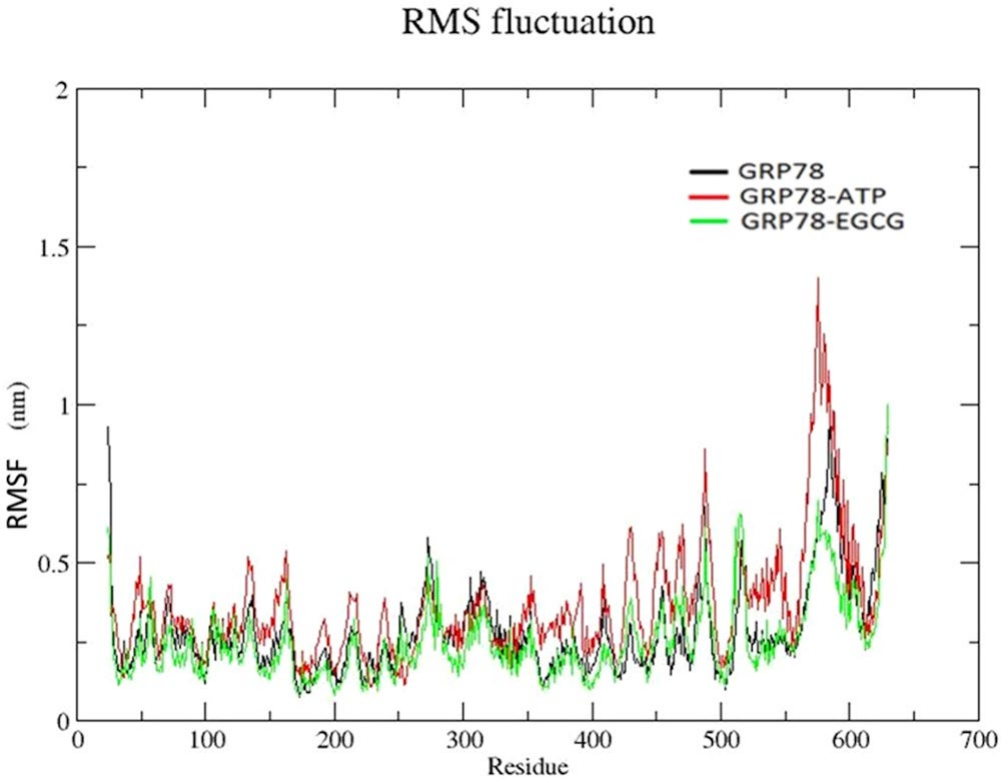
Root mean-square fluctuation (RMSF) plot for apo GRP78 protein (black), ATP-bound system (red) and EGCG-bound system (green).

In order to observe mobility of different residues, RMSF plots were generated for all the 3 simulations (Fig. 4). The three structures show RMSF values in the range of 0.2–0.8 nm, 0.2–1.3 nm, 0.2–0.6 nm for apo state of protein, GRP78-ATP and GRP78-EGCG complexes, respectively. As observed, GRP78, when complexed with ATP, shows the highest mobility of residues throughout. Apo state shows lesser mobility than ATP-bound state and in EGCG-bound form, it shows the least mobility of the residues. We have neglected 10 residues from both the ends of N terminal and C terminal region. These terminal end residues often show high mobility because they reside at the ends of protein molecule. From this plot, we can conclude that binding of EGCG inhibitor reduces the mobility of the residues more as compared to the ATP-bound state.

### Protein compactness and folding

**Figure 5:**
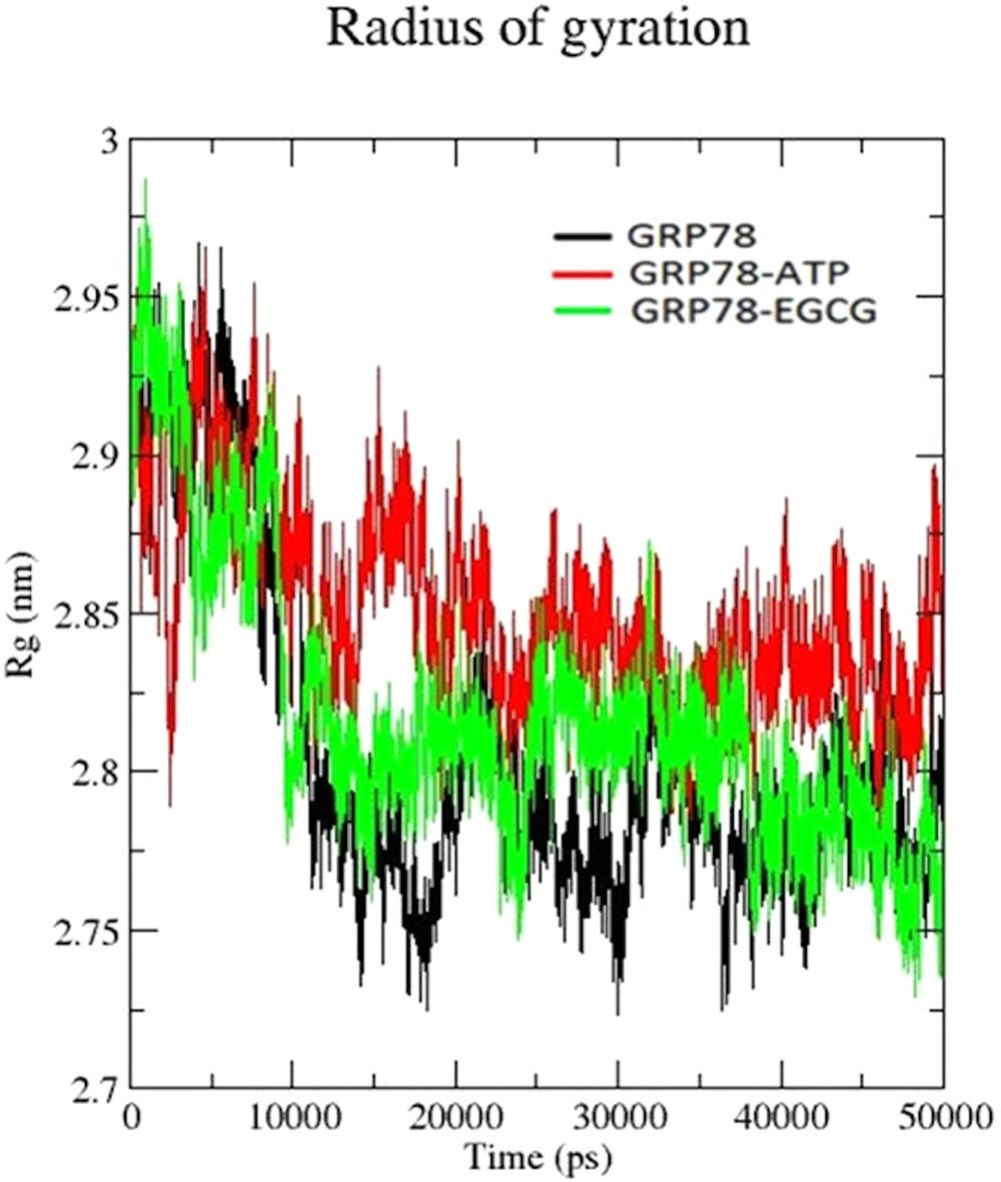
Radius of gyration (R_g_) *vs.* MD simulation time plot for apo GRP78 protein (black), ATP-bound system (red) and EGCG-bound system (green).

For a protein, a measure of its structural compactness is its radius of gyration (R_g_). A relatively steady value of R_g_ over time shows that a protein is stably folded. To check this, radius of gyration vs. time graphs have been plotted for all the three structures (Fig. 5). After initial fluctuations, the R_g_ in both apo state and EGCG-GRP78 complex maintains a relatively steady value with an average value of 2.78 nm, this starts from 10 ns till the end of simulation time. In contrast, binding of ATP shows a different scenario. In the latter case, the protein is unable to maintain a steady state of R_g_ till 25 ns (average of 2.83 nm). After 25 ns, the fluctuation is comparatively steady, but still with higher fluctuation as compared to other two simulations. This indicates that binding of ATP unfolds the protein further, thereby helping in proper functioning of GRP78, as a slightly unfolded form of this protein is required to maintain its activity^11,16^. Binding of EGCG inhibitor gradually folds the protein, leading to malfunctioning of GRP78. This is also consistent with our observations from previous paper^11^.

### Hydrogen bonds

**Figure 6:**
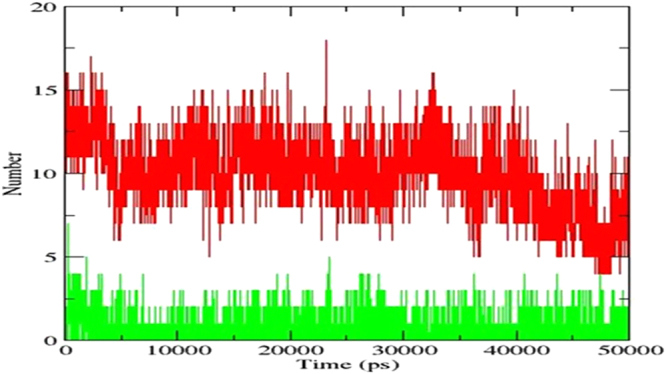
Number of hydrogen bonds *vs.* MD simulation time for ATP-bound system (red) and EGCG-bound system (green).

As the number of hydrogen bonds is related to binding strength, a graph was plotted to find out the number of hydrogen bonds between the ligands and the protein (Fig. 6). It was found that, between ATP and protein, the number of intermolecular hydrogen bonds with a maximum value is an average of 15 and only one conformation has more number of hydrogen bonds,i.e., 18. In the case of GRP78-EGCG complex, the number of intermolecular hydrogen bonds with a maximum value is an average of 5 and only one conformation has more number of hydrogen bonds, i.e., 7, during the trajectory period. The number of hydrogen bonds were consistent with those found in the crystal structure and docking studies. From these observations, although ATP appears to be more tightly bound to the protein molecule, EGCG binding strength is reduced in comparison, and may form transient interactions. It seems that for EGCG inhibitor to be effective, a steady concentration of EGCG will be required to be maintained in order to be kept bound to the target, and to further prevent ATP from binding.

#### Movement of SBDα and SBDβ sub-domains upon EGCG binding

As mentioned before, in GRP78, residues 419–633 belong to the SBD and residues from 25–408 belong to NBD. In SBD, residues 419–529 comprises of SBDβ sub-domain and residues 529–633 occupy the SBDα sub-domain, which acts as the lid for the SBDβ sub-domain. For the substrate to bind properly to the SBD, the movement of SBDα as a lid over SBDβ plays a crucial role. If the movement is hindered, the functioning of GRP78 gets affected drastically, and the substrate is released even before it gets folded in its functional form. From the RMSF graphs, observing more closely, we can compare the mobility of SBD consequent upon binding of ATP and inhibitor EGCG. We can see that, protein in the complex with ATP shows the highest mobility of 1.4 nm in residue range of 529–633. This range of residues in apo state shows highest fluctuation till 0.9 nm only and in complex with EGCG inhibitor, it shows the lowest fluctuation (about 0.3 nm). From our observations, in the apo state, the mobility of SBDα sub-domain is less compared to that of ATP-bound form. When ATP binds, the mobility of the residues increases. The binding of EGCG reduces mobility to even lesser value than that of the apo state. As a result, the movement of SBDα subdomain is hindered, preventing its normal role as a lid and GRP78 may not be able to carry out its function, therefore.

### SBDα and SBDβ subdomain interactions: Distances and angles

To more clearly observe the movement of the SBDα domain, Calpha-Calpha atom distances between two residue points in SBDα and SBDβ were calculated at each time point for 50 ns. In SBDα, Lys587 residue and in SBDβ, Ala486 residue was selected based on our observations in RMSF plots. In this plot, in SBDβ subdomain, residue range 485–490 shows the maximum fluctuation. Likewise, in SBDα sub-domain, residue range showing the maximum fluctuation is 585–595. As visualized by PyMol, we can see that when SBDα lid closes over SBDβ, residue Ala486 in SBDβ sub-domain and residue Lys587 in SBDα sub-domain reside at the meeting points where the lid meets SBDβ sub-domain. These two points have the maximum distance between SBDβ and SBDα than any other points when the lid opens and minimum when the lid closes. As we wanted to study the opening and closing of lid due to domain movements before and after the substrate/inhibitor is bound, we took these residues to measure the distance at different time periods in different conformations. Using the PyMol visualization tool, final .pdb file was split into 50 parts representing each ns time. Distances were calculated at each of the total 50 ns timepoint for all the three structures. Finally, a line graph was constructed.

**Figure 7:**
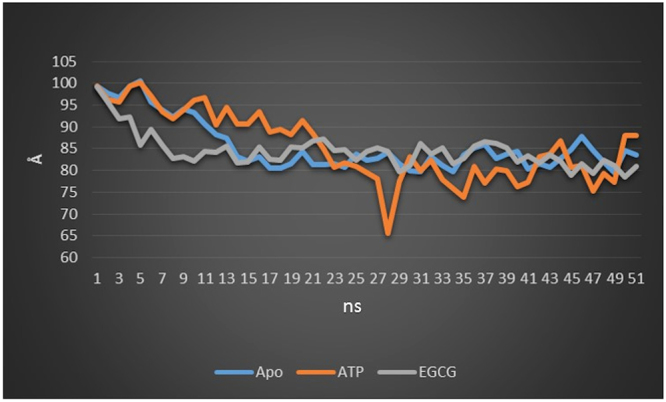
Graph representing Calpha-Calpha distances between Lys587 residue and Ala486 residues at each point of time. Apo state plot is shown in blue, ATP-bound state in orange and EGCG-bound state in grey.

From the graph, (Fig. 7) we can see that, distance between the two residues differs at each ns, in all the three structures. In apo state, distance between the two residues reaches at a constant level after 15 ns with an average value of 85 Å. After that, it does not show any increase. In EGCG -bound state, it arrives at a constant value earlier, after 9 ns. This plot too, shows a constant average value of 85 Å, with no further increase in value with time. However, the time taken to arrive at a constant level is lesser than that of apo state of the protein. Protein in complex with ATP shows a wider range of fluctuation of distances in the range of 65 Å–100.2 Å, with a minimum distance of 65.6 Å and a maximum value of 100.2 Å. At the beginning, it shows a distance of 99.3 Å and with time, it decreases to 65.6 Å (28^th^ ns). As the simulation continues, the distance again increases. This indicates the widespread movement of SBDα subdomain consequent to the binding of ATP molecule and its ability to, act as a lid for SBDβ subdomain, in conformity with earlier studies on the mechanism of action of HSP70 protein. From this plot, we can observe that binding of ATP leads to increased movement of SBDα. In contrast, binding of EGCG reduces its mobility, thereby slighlty closing the lid over SBDβ. It is perceived that as a result, the substrate may not be able to access SBD, effectively inhibiting GRP78 function. Compared to ATP bound state, apo state also shows lesser movement of SBDα.

**Figure 8:**
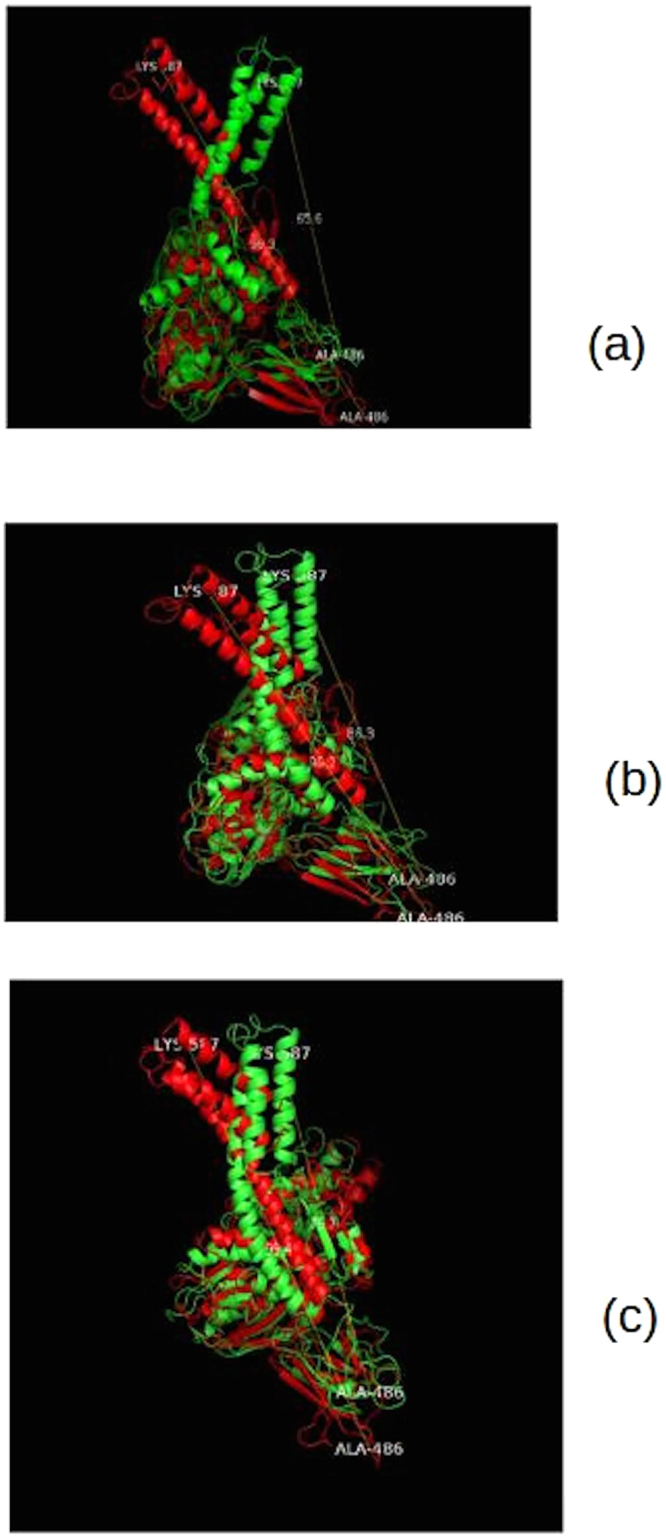
Comparison of differences in conformations measured using distances at 1^st^ ns and 28^th^ ns for ATP-bound and 1^st^ and 31^st^ ns for EGCG-bound system. Initial conformation is depicted in red and maximum deviation from the initial conformation is depicted in green color in all the three instances. (**a**) Deviation of ATP-bound protein. (**b**) Deviation of EGCG-bound protein. (**c**) Deviation of apo state of protein.

From Fig. 8, we can see that not only the SBDα domain moves upon ATP and EGCG binding to NBD, but even the SBDβ subdomain shows movement towards SBDα. We can cross check this with RMSF graph. Fluctuation of residues 419–529 is higher compared to the other parts of the protein (such as NBD). Binding of EGCG reduces the mobility of these residues.

As has already been mentioned above, in open conformation, the angle between SBDα and SBDβ is much larger than that of the closed one. To check this in our simulations, apart from Lys587 and Ala486 residues, a third residue, Arg528 was also selected in order to calculate the angle difference at each nanosecond. This residue is in middle of the region connecting SBDα subdomain to SBDβ subdomain. Angles for each nanosecond were calculated, and finally a line graph was constructed.

**Figure 9:**
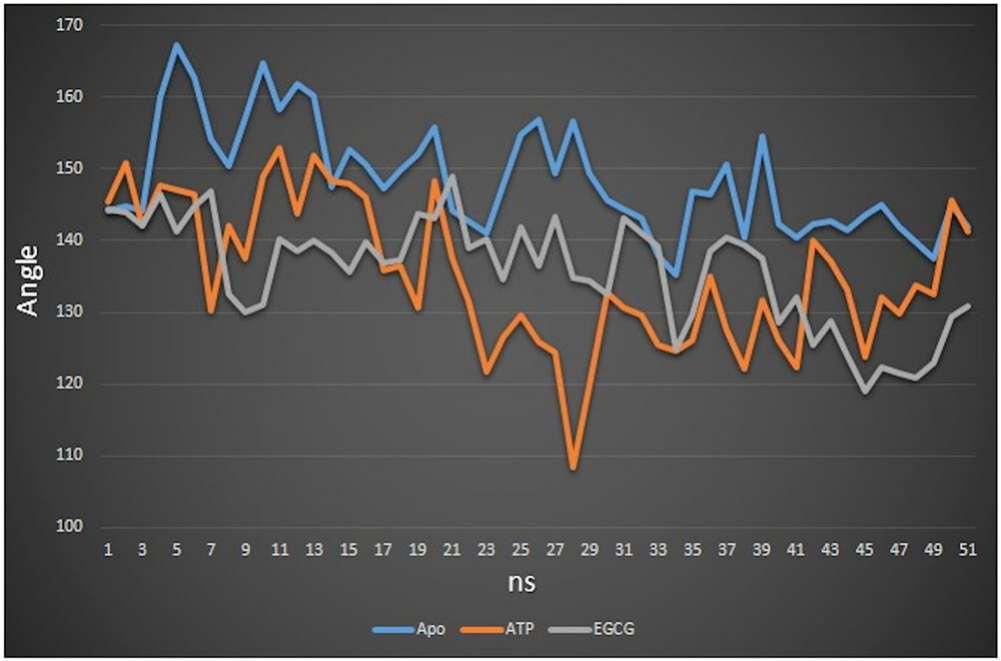
Graph representing Calpha-Calpha-Calpha atom angles between Lys587 residue, Arg528 and Ala486 residues at each point of time. Apo state plot is shown in blue, ATP-bound state in orange and EGCG-bound state in grey.

**Figure 10:**
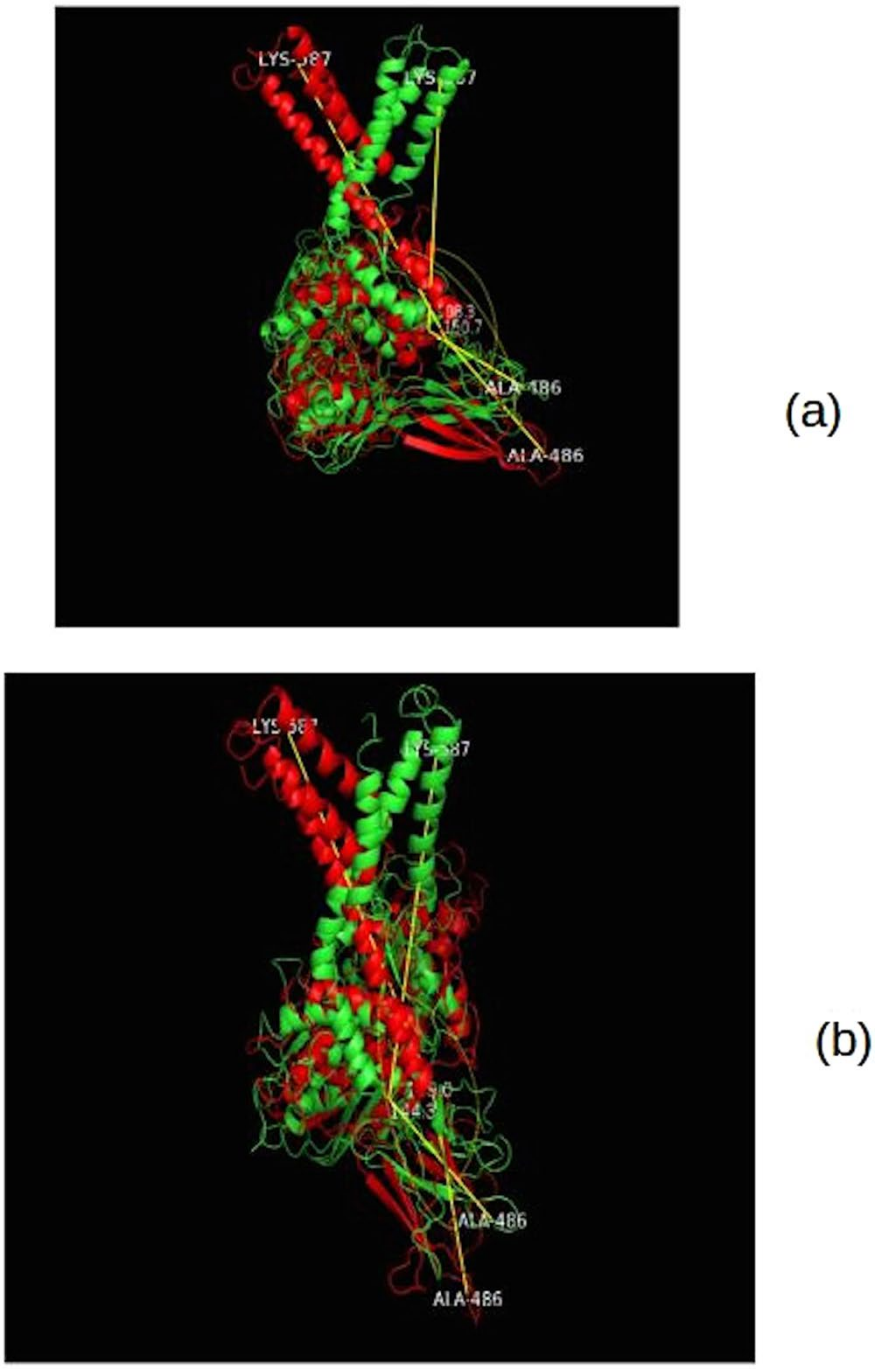
Comparison of differences in conformations measured using angles at 1^st^ ns and 28^th^ ns for ATP-bound and 1^st^ and 31^st^ ns for EGCG-bound system. Initial conformation is depicted in red and maximum deviation from the initial conformation is depicted in green color in both instances. (**a**) Deviation of ATP-bound protein. (**b**) Deviation of EGCG-bound protein.

We observed that the angle values differ at each nanosecond in case of all the three structures (Fig. 9). In this graph, we can observe that ATP-bound protein, again, shows more fluctuation of values. From 1–28 ns, showing an initial decrease, it starts increasing ending up at similar place when it started. In EGCG complex simulation, the angle values show an initial increase, decreasing gradually throughout the simulation time, and the pattern throughout is little bit similar to that of apo state. This further indicates that the movement of SBDα increases in the ATP-bound state and gets hindered with the binding of EGCG inhibitor. The latter may lead to the situation where peptide substrate is unable to associate with SBD properly. The maximum deviation of angle with respect to the initial state can be observed in Fig. 10. These patterns from measuring angles between three residues are similar to the patterns produced on measuring distances between two residues.

Our results on apo state and ATP-GRP78 complex simulations are similar to some of the previous works done on HSP70 proteins. Crystal structures and NMR analysis have revealed structures of three different functional states: ADP-bound^20,21^, ATP-bound^22,23^ and ADP/substrate-bound states^23^. In undocked state (ADP-bound), SBD and NBD have been found to behave independently and linker does not interact with NBD, while SBDα lid is linked to the SBDβ subdomain. With the binding of ATP, it results in the formation of NBD-SBDβ favorable structure, due to the linker interaction with NBD, while the interaction of SBDα with SBDβ is not favored.

After ATP gets bound, it communicates with the four subdomains of NBD and helps in binding of interdomain linker to NBD adopting a beta-strand conformation, which results in bringing NBD and SBD together^24–26^. Further, previous work shows that in the domain docked state, dissociation of SBDα from SBDβ results in decrease of substrate affinity (10 folds) and increase substrate binding/release kinetics^27–29^. Due to the movements of SBDα as a lid over SBDβ, it changes the substrate binding/releasing kinetics. In our molecular dynamics studies, we have observed the movement of SBDα subdomain, with respect to SBDβ, in the apo state and the complex structure with ATP. This is also the case with GRP78-EGCG simulations which are more similar in overall pattern to that of the apo state in each of the parameters tested. Our analyses show that not only the SBDα undergoes conformational changes, the intradomain rearrangements in the SBDβ subdomain too occur during the binding of ATP as well as EGCG. This is in agreement to the previous work which has been done on SBDβ subdomain. It has been found that two sub-subdomains of SBDβ, sub-subdomain I and sub-subdomain II (sub-subdomain I comprises residues 420–437 and 456–485, sub-subdomain II comprises of residues 400–419 and 440–455) display a semi rigid body seesaw-like rearrangement^28^. While in ATP-bound state, the SBD is more mobile and can open and close easily to facilitate substrate peptide access, in EGCG bound forms, the mobility of residues is reduced which may make it difficult for substrate peptides to bind SBD. This may be the mode through which EGCG may inhibit GRP78.

In sum, our paper strives to provide a structural insight into the mechanistic basis of action of inhibitor EGCG on GRP78 as compared to ATP-bound and apo state. From previous work in our lab, we have found that EGCG changes NBD domain conformation^11^. This paper goes further to explore SBD domain conformational changes consequent upon NBD domain structural changes. Powerful molecular dynamics studies have helped us elucidate large-scale conformational movements of SBD emerging as a result of the binding of the EGCG inhibitor to NBD and the interaction effects traveling onwards through linker domain to influence SBD movements.

## Conclusions

GRP78 which is overexpressed in glioblastoma has been found to play a role in its development and proliferation. Despite the discovery of inhibitors for GRP78, the effect of these inhibitors on the protein conformational changes and structural basis of their action have not been elucidated in detail. Molecular docking and molecular dynamics studies using full-length GRP78 in this paper show that binding of EGCG stabilizes the protein faster than the apo state of protein. ATP binding increases the time taken to stabilize the protein. It is further observed that binding of ATP increases the movement of SBDα subdomain. This movement is required to help substrate peptides bind properly to the SBDβ. At the same time, binding of EGCG inhibitor hindered the movement of SBDα subdomain, which in turn, may reduce the binding of substrate peptide to SBDβ. EGCG binding folds the protein steadily while binding of ATP unfolds the protein. A slightly unfolded form is the native functional form of GRP78. So, as a result of binding EGCG and large-scale movements traveling from NBD to SBD, GRP78 will not be able to carry out its proper functions in view of global conformational changes and hindrance of SBD movements. This may be one of the likely mechanisms by which EGCG inhibitor acts on full-length GRP78 protein, thereby targeting glioblastoma as well as other types of tumors.

## Electronic supplementary material

Supplementary Information
