# Supplementary material for: Glucose-regulated protein 78 substrate-binding domain alters its conformation upon EGCG inhibitor binding to nucleotide-binding domain: Molecular dynamics studies
Source: Sci Rep. 2018 Apr 3;8:5487. doi: 10.1038/s41598-018-22905-6 (PMC5882873; doi:10.1038/s41598-018-22905-6)
Supplement: Supplementary file 1 — Supplementary Information [file 41598_2018_22905_MOESM1_ESM.pdf]

## Supplementary Data

# Glucose-regulated protein 78 substrate-binding domain alters its conformation upon EGCG inhibitor binding to nucleotide-binding domain: Molecular dynamics studies

K. R. D. Sagara N. S. Gurusinghe, Aanchal Mishra and Seema Mishra

**Material included:**

Figure S1

Figure S2

Figure S3

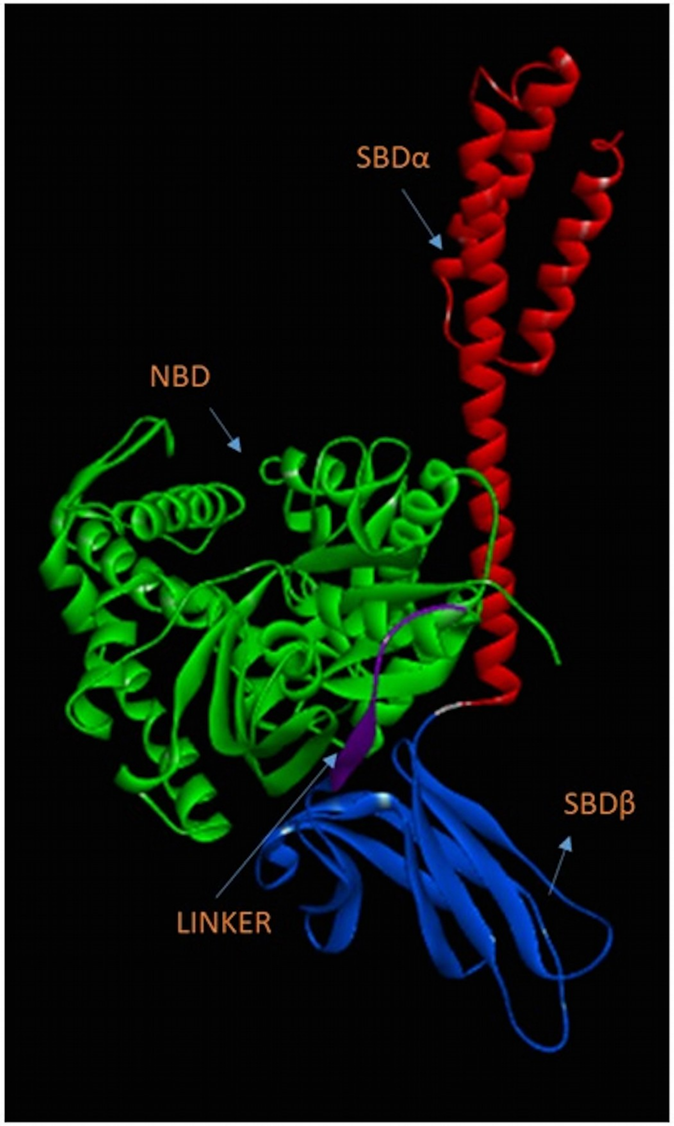


**Figure S1**. Structure of GRP78 protein (PDB ID:5E84) as a cartoon representation. Subdomains are colored as follows: Nucleotide-binding domain (Green), Linker region (purple), Substrate-binding subdomainβ (blue), and Substrate-binding subdomainα (red).

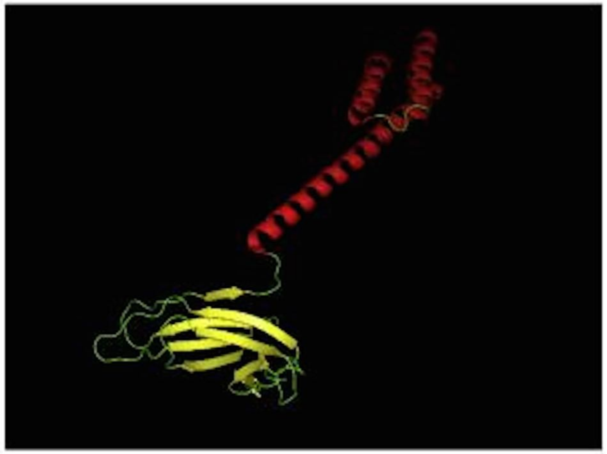

(a)

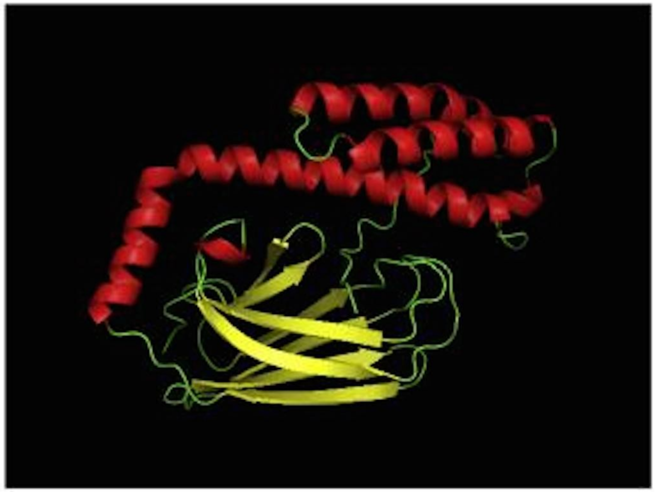

(b)

**Figure S2.** Substrate-binding domain conformations in open (a) and closed (b) forms of GRP78. Taken from PDB IDs: 5E84 for full length form (NBD is deleted for the sake of clarity and comparison purpose) and 5E85, for isolated SBD domain, respectively.

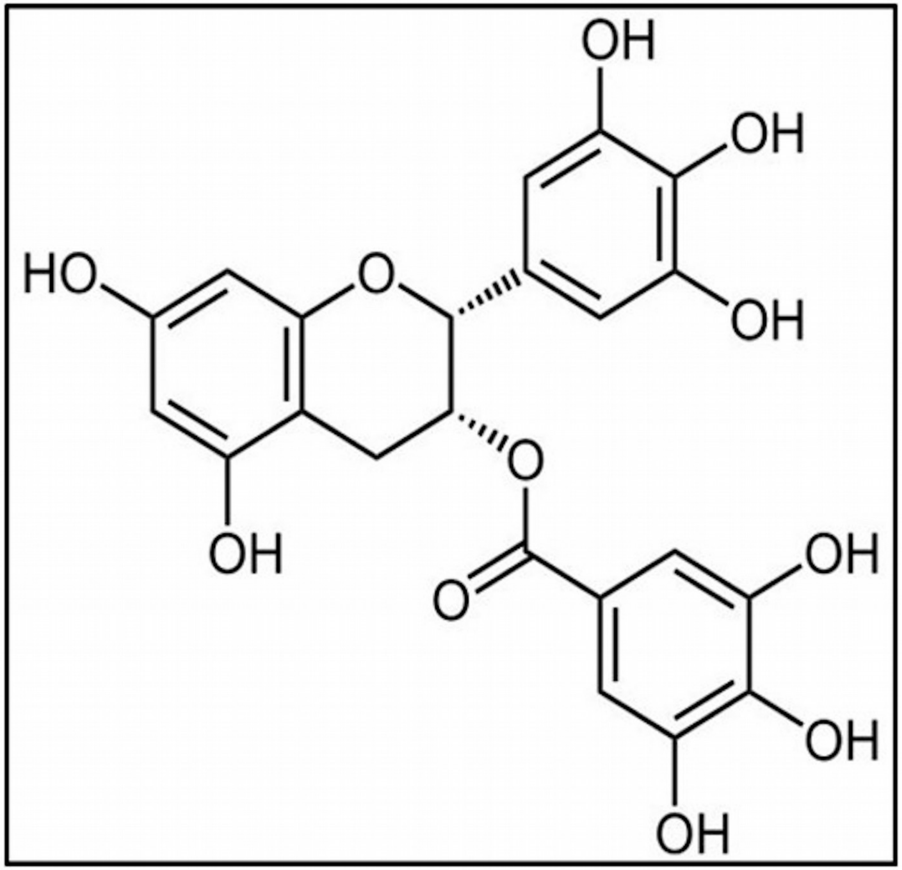


**Figure S3.** 2D-Structure of (-)-Epigallocatechin gallate (EGCG) inhibitor.
